# Vitamin D–AMP axis in host defense against fungal infections

**DOI:** 10.3389/fnut.2026.1807743

**Published:** 2026-06-04

**Authors:** Nuraly S. Akimbekov, Ilya Digel, Svetlana K. Sakhanova, Kuanysh T. Tastambek, Dinara K. Sherelkhan, Mohammed S. Razzaque

**Affiliations:** 1 International Center for Islamic Science and Innovation, Al-Farabi Kazakh National University, Almaty, Kazakhstan; 2 Ecology Research Institute, Khoja Akhmet Yassawi International Kazakh-Turkish University, Turkistan, Kazakhstan; 3 Scientific-Practical Center, West Kazakhstan Marat Ospanov Medical University, Aktobe, Kazakhstan; 4 Institute for Bioengineering, Aachen University of Applied Sciences, Jülich, Germany; 5 Department of Medical Education, School of Medicine, University of Texas Rio Grande Valley (UTRGV), Edinburg, TX, United States; 6 Faculty of Medicine, Universiti Kuala Lumpur Royal College of Medicine Perak (UniKL RCMP), Ipoh, Perak, Malaysia

**Keywords:** antimicrobial peptide, cathelicidin, defensins, fungal infection, innate immunity, VDR, vitamin D

## Abstract

Vitamin D–mediated regulation of antimicrobial peptides (AMPs) is an important focus in innate immunology and is aimed at elucidating the role of vitamin D in enhancing antimicrobial defense. AMPs are short protein chains that serve as a first line of defense against invading pathogens, including fungi, bacteria and viruses. Unlike conventional antibiotics, AMPs are produced endogenously and are less likely to induce antimicrobial resistance, making them promising candidates for treating infections caused by drug-resistant pathogens. Studies indicate that optimal vitamin D levels are essential for activating antimicrobial pathways and regulating AMPs that target multiple fungal pathogens. This article summarizes recent findings on vitamin D-induced AMPs in the context of invasive fungal infections. It also distinguishes vitamin D as a host immune modulator from vitamin D3 as a putative active antifungal compound, given that direct antifungal use is limited by supraphysiologic dosing requirements, pharmacologic impracticality, and risks of hypercalcemia and hyperphosphatemia, especially in patients with granulomatous diseases. Model limitations and species differences are also discussed, including primate-specific CAMP vitamin D response element regulation, which constrains direct translation of rodent vitamin D-to-LL-37 findings to human fungal disease. Current global fungal priority frameworks and resistance surveillance support emphasizing *Candida*, *Aspergillus*, and *Cryptococcus* in this review of invasive fungal disease and translational host-defense evidence, underscoring the relevance of these pathogens.

## 1 Introduction

The incidence of fungal infections, particularly severely invasive infections, has risen significantly in recent decades because of several factors: a growing population of immunocompromised individuals, widespread use of broad-spectrum antibiotics and chemotherapy, increased organ transplantation, and the increasing prevalence of health conditions such as diabetes and COVID-19 (1). This trend is concerning, as it is coupled with the emergence of drug-resistant fungal strains and limited treatment options, leading to a substantial global health burden (2). In line with updated global-priority frameworks and recent resistance surveillance, this review places primary emphasis on *Candida, Aspergillus,* and *Cryptococcus* as the most clinically consequential invasive fungal pathogens, whereas *Trichophyton* and other resistant dermatophytes are discussed more selectively because their translational AMP literature remains less developed, and this review is centered on invasive fungal disease (3–5).

Recent studies have estimated that more than 3.75 million deaths annually are linked to invasive fungal diseases (6), suggesting that the true global burden of fungal infections is substantially greater than previously thought (3), with approximately 6.55 million people affected each year and a significant number of deaths (approximately 2.55 million) directly attributed to these diseases. A drastic increase in antifungal resistance to azoles and echinocandins, which remain cornerstone treatments for many common fungal infections, has been reported (7–9). Resistance often develops through genetic changes in fungi, such as mutations in drug targets or increased drug efflux. This growing problem poses a serious challenge to public health, limiting treatment options and increasing the risk of adverse outcomes for patients, particularly those with invasive candidiasis and mold disease (10). The development of antifungal drugs is further complicated by the close evolutionary relationship between fungi and humans. Unlike bacteria, fungi share many fundamental eukaryotic features with human cells, including metabolic pathways and plasma membrane composition, resulting in the formation of fewer pathogen-specific drug targets (11).

Interest in antimicrobial peptides (AMPs) as antifungal agents is increasing because of the limited effectiveness of current treatments and the global crisis of antifungal resistance (9, 12, 13). AMPs are promising alternatives or supplements to existing antifungals because they are naturally occurring, possess broad-spectrum antimicrobial activity, and are less likely to induce resistance than traditional drugs are (14). Growing evidence suggests that vitamin D increases innate immunity by regulating the production of AMPs (15–17). The active form of vitamin D, 1,25(OH)_2_D_3_, can induce the expression of AMPs such as cathelicidin and defensin, thereby potentially enhancing host antifungal defense (18). However, this host-directed immunomodulatory role should be distinguished from the separate hypothesis that native vitamin D3 itself can function as a directly administered antifungal agent. These are not interchangeable claims: the former concerns the regulation of host innate immunity, whereas the latter requires independent evidence for achievable antifungal exposure, efficacy, and safety.

This narrative review synthesizes current evidence on the vitamin D–AMP axis in innate immunity and elaborates the role of vitamin D as an immune modulator from that of vitamin D3 as a potential direct antifungal agent. Pathogen prioritization aligns with WHO fungal priority frameworks and contemporary resistance surveillance, ensuring a clinically relevant and globally consistent context.

## 2 Literature search criteria

### 2.1 Narrative review approach and pragmatic search strategy

This article was prepared as a narrative review rather than a systematic review because the available evidence spans mechanistic studies, cell-based experiments, animal models, and a limited number of clinical reports. For pragmatic transparency, PubMed/MEDLINE, Scopus, Web of Science, and Google Scholar were searched for English-language publications from January 2000 to April 2026, and reference lists of key papers and relevant reviews were hand-searched for additional studies. A focused citation update during revision was also used to incorporate newly published reviews relevant to vitamin D in infectious diseases. The search terms included combinations of “vitamin D,” “1,25(OH)_2_D_3_,” “25(OH)D_3_,” “vitamin D receptor,” “antimicrobial peptides,” “AMPs,” “cathelicidin,” “LL-37,” “defensins,” “fungal infection,” “*Candida*,” “*Aspergillus*,” “*Cryptococcus*,” “*Trichophyton*,” “dermatophytes,” “autophagy,” and “innate immunity.” Priority was given to studies directly examining vitamin D-dependent AMP regulation and antifungal host-defense outcomes. During final reference updating, preference was given to the literature published within the past 5 years (2021–2026) for the introduction, discussion, epidemiology, resistance, and translational framing, whereas older studies were retained only when they described seminal mechanisms or foundational observations that remain directly relevant. When the findings were conflicting, they were interpreted in light of the study design, model system, vitamin D form and dose, pathogen species, and tissue context, and unresolved inconsistencies were retained as knowledge gaps rather than forced into consensus. Accordingly, this manuscript should be interpreted as a qualitative narrative synthesis rather than a PRISMA 2020-compliant systematic review. In the synthesis below, the antifungal evidence is additionally tiered as mechanistic, animal model, observational human, and randomized trial evidence, and clinical inferences are restricted accordingly.

### 2.2 Literature selection criteria and evidence prioritization

Articles were deemed eligible for inclusion if they addressed at least one of the following criteria: (1) vitamin D status, signaling, metabolism, receptor activity, or supplementation in relation to fungal infection; (2) vitamin D-dependent regulation of antimicrobial peptides or innate immune pathways directly relevant to fungal host defense; (3) antifungal activity, fungal pathogenesis, or translational implications involving LL-37, defensins, or related host-defense peptides; or (4) clinically significant fungal pathogens prioritized in this review. Basic-science studies outside fungal systems were included only when they provided the essential mechanistic context necessary for interpreting fungal findings, such as VDR-CAMP signaling, AMP effector biology, or species-specific regulatory constraints. Studies were deprioritized or excluded if they were duplicate reports, conference abstracts lacking sufficient data, non-English publications, articles focused exclusively on nonfungal infectious outcomes without clear relevance to fungal host defense, or articles whose content did not materially inform the review questions. Evidence was prioritized according to translational proximity: randomized trials and human clinical studies informed clinical implications; observational studies described associations and hypothesis-generating signals; animal models assessed *in vivo* plausibility and dose-dependent effects; and mechanistic or cell-based studies elucidated pathways and identified targets rather than supporting stand-alone therapeutic claims. This approach ensured that clinical interpretation remained aligned with the strongest available evidence while integrating mechanistic insights relevant to fungal pathogenesis and AMP biology.

### 2.3 Pathogen prioritization

Because this manuscript is framed around invasive fungal disease and translational host-defense relevance, pathogen emphasis was not determined by literature availability alone. It was anchored to current global priority-setting and resistance surveillance. In the WHO fungal priority pathogens list (FPPL), *Cryptococcus neoformans, Candida auris, Aspergillus fumigatus,* and *Candida albicans* are placed in the critical-priority group, which supports the main focus of the review on *Cryptococcus, Aspergillus,* and *Candida* as the most clinically consequential organisms in this field (19). Within that focus, *Candida* is discussed broadly because much of the vitamin D-AMP literature concerns *C. albicans,* whereas the present-day clinical urgency of the genus is further reinforced by surveillance showing that *C. auris* spreads in healthcare settings and can cause severe multidrug-resistant illness (20). Similarly, *Aspergillus* is emphasized because *A. fumigatus* is both a WHO critical-priority pathogen and the leading cause of invasive mold infections, with emerging azole resistance recognized as a growing global public health concern (19, 20). *Trichophyton* and other dermatophytes remain important because ringworms have a very large worldwide burden, and resistant diseases, including the emergence of resistant *Trichophyton* lineages, are increasingly recognized; however, these diseases are treated more selectively here because this review focuses on invasive fungal infection and because the vitamin D-AMP evidence base is less clinically developed for dermatophytes than for *Candida* and *Aspergillus* (21, 22). Given this clinically prioritized context, the next section presents the essential background needed to interpret the subsequent fungal-specific and translational evidence. This structure focuses the review on its main question rather than on the broader phenomenon.

## 3 Vitamin D, antimicrobial peptides and fungal host defense

Vitamin D is acquired through dietary intake or synthesized in the skin following UV-B exposure (23–27). The two primary forms, vitamin D2 (ergocalciferol) and vitamin D3 (cholecalciferol), undergo sequential hydroxylation to form 25(OH)D and subsequently the metabolically active 1,25(OH)_2_D_3_. A key consideration is that epithelial and immune cells express vitamin D receptors (VDRs) and can locally activate or respond to vitamin D. This links vitamin D status to innate immune defense, extending its role beyond calcium–phosphate homeostasis (28–33). Through VDR-dependent transcription and associated signaling pathways, vitamin D modulates AMP expression, phagocyte function, autophagy, barrier integrity, and inflammatory responses (34–42). Given the established vitamin D framework, the discussion now focuses on antimicrobial peptide families most pertinent to fungal host defense, as these peptides constitute the primary proposed effector link between vitamin D signaling and subsequent antifungal responses.

**Figure 1:**
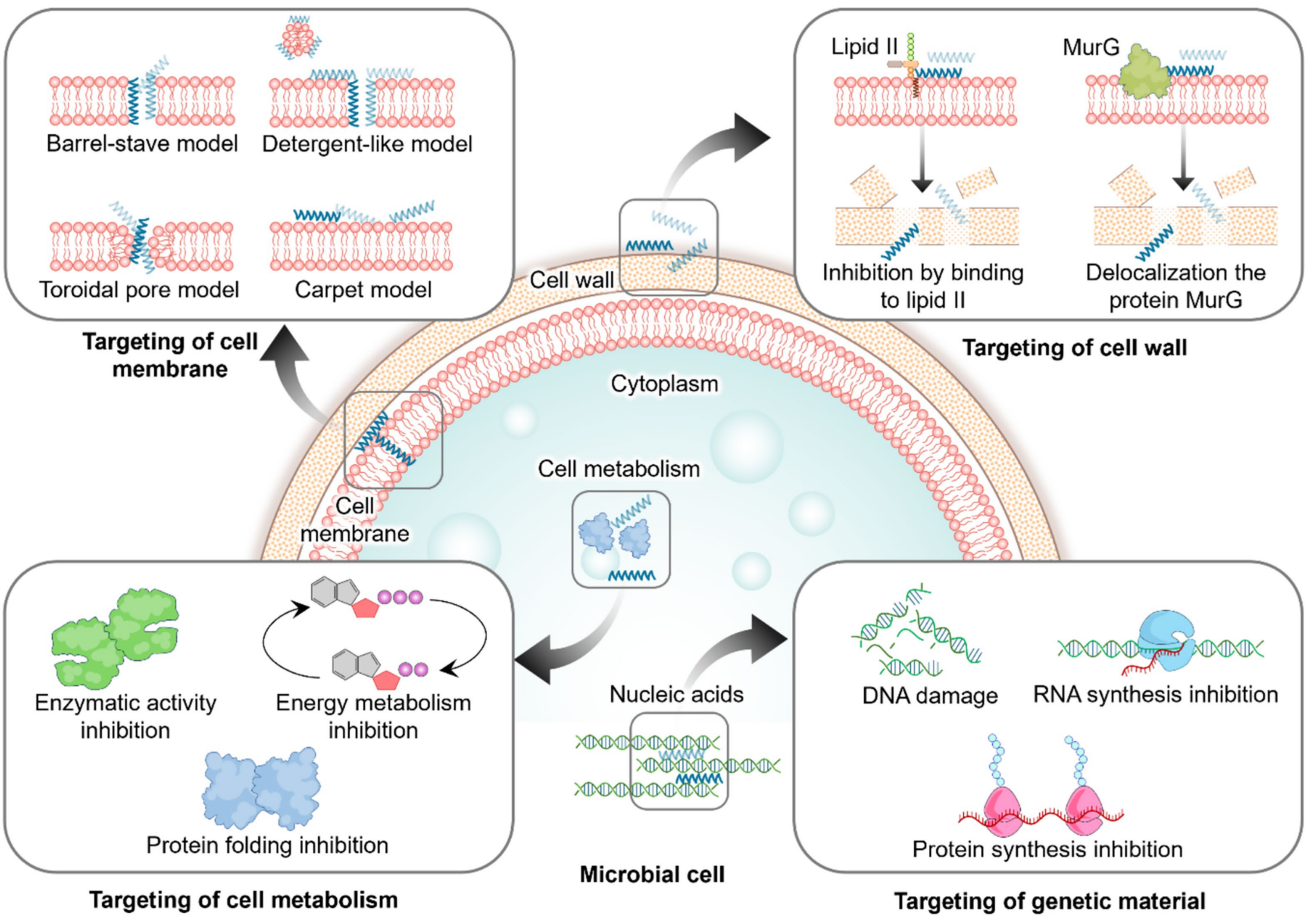
General antimicrobial mechanisms of AMPs (114, 140, 159, 160).

AMPs are small, predominantly cationic effector molecules produced by epithelial cells and leukocytes (43–45). Among human AMPs, defensins and the cathelicidin LL-37 are most relevant to this review, as they are frequently discussed in mechanistic, animal model, and translational studies (46–52). These peptides function by disrupting microbial membranes, targeting intracellular components, influencing biofilm formation, and modulating leukocyte recruitment and inflammatory signaling (Figure 1) (49, 53–58). In this context, the host-defense actions relevant to fungal pathogens are more informative than broad structural classifications or comprehensive AMP catalogs (59–65). Therefore, the following discussion emphasizes AMP–fungus interactions and their translational implications.

## 4 Vitamin D: immunomodulator versus direct antifungal effects

In this section, we emphasize two important issues: the first issue is whether vitamin D significantly modulates antifungal host defense through antimicrobial peptides and related innate pathways. The second issue, discussed separately below, is whether vitamin D3 has a plausible role as a directly administered antifungal compound.

### 4.1 Vitamin D as an immune modulator of antifungal host defense

At the host level, vitamin D, especially its active form 1,25(OH)_2_D_3_, functions primarily as an immune modulator that can strengthen innate defense in different organs by promoting the expression of AMPs, including LL-37 and human *β*-defensins (66). In this context, vitamin D does not act as a conventional antifungal drug; rather, it shapes barrier, inflammatory, and phagocyte responses that may secondarily improve antifungal control.

Epithelial cells in the intestinal and respiratory systems secrete AMPs that disrupt microbial cell membranes (67). They are particularly important in regions of the small intestine and lung alveoli that lack a significant mucus barrier, where they act in concert with a mucus coating to provide broad protection against microbial invaders (68). Specialized Paneth cells in the intestinal crypts of Lieberkühn secrete AMPs, whereas in the lungs, AMPs are delivered to the airway surface liquid, a mucin-rich fluid, and are produced by respiratory submucosal glands (69).

**Figure 2:**
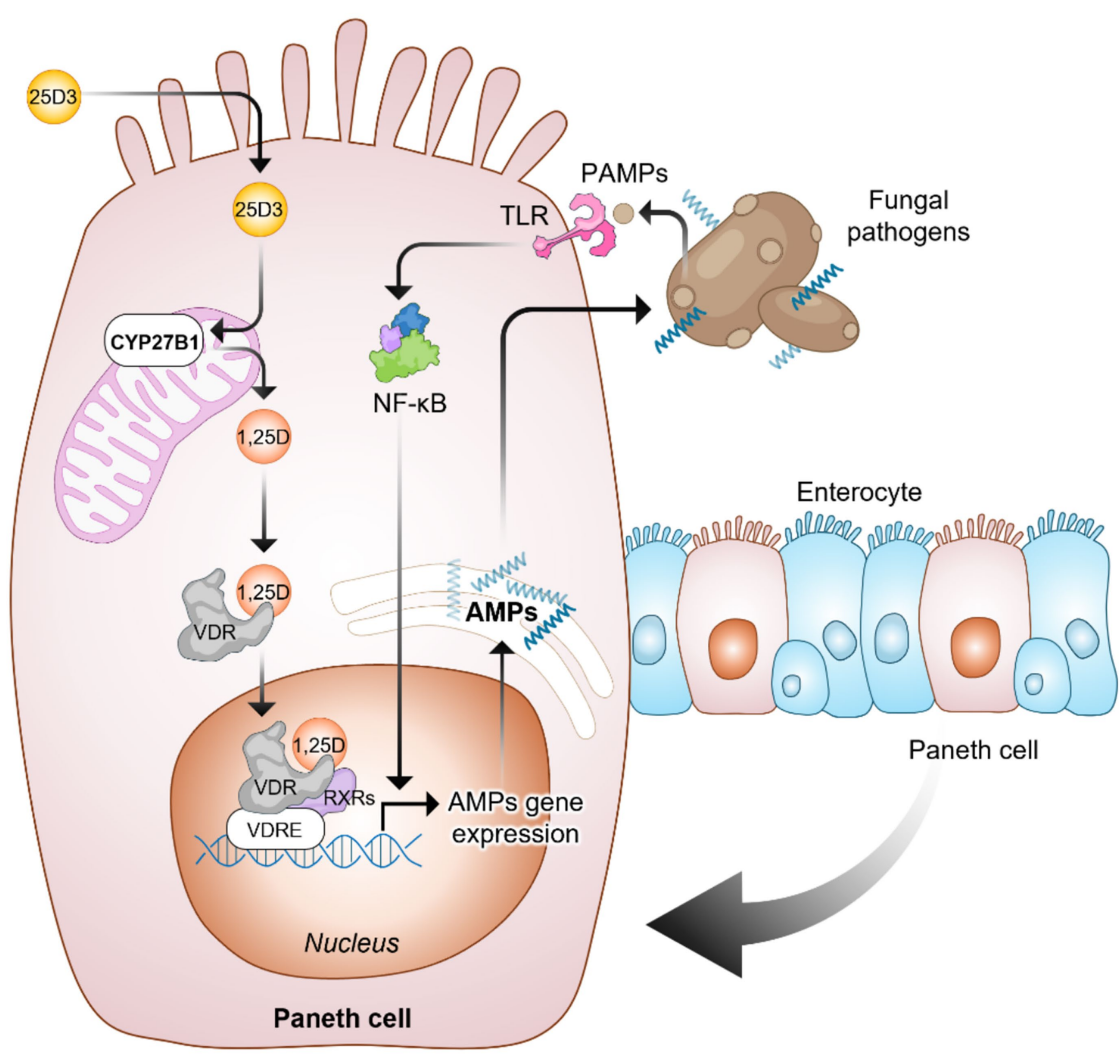
Induction of AMPs by vitamin D in epithelial cells. PAMPs, Pathogen-associated molecular patterns. See further explanations in the text.

The vitamin D activation pathway begins in epithelial cells, where inactive circulating 25(OH)D_3_ enters immune or epithelial cells and is converted into hormonally active 1,25(OH)_2_D_3_ by the enzyme CYP27B1 (Figure 2). This active form binds to the VDR, which then partners with RXR to form the VDR-RXR complex. This complex attaches to specific DNA sequences, namely, VDREs, turning on genes that encode AMPs. In primates, the CAMP gene (LL-37) contains a strongly functional VDRE in its promoter and is directly induced by vitamin D, whereas this specific regulatory architecture is not conserved in standard rodent models (70–72). The DEFB4 gene (hBD-2) usually requires a combination of inflammatory signals, such as those mediated by the NF-κB signaling pathway, for full expression. When microbial pathogens are detected, TLR signaling stimulates the expression of both VDR and CYP27B1, resulting in potent and localized antimicrobial responses (70).

Vitamin D/VDR signaling culminates in the production of hCAP-18/LL-37 through *CAMP* gene upregulation. hCAP-18 is a propeptide composed of two main domains: a conserved N-terminal cathelin domain (which functions as a cysteine protease inhibitor with broad-spectrum antibacterial actions) and a variable C-terminal LL-37 domain (37-amino acid sequence with an amphipathic *α*-helix structure, which is essential for its ability to disrupt cell membranes and exert its antimicrobial functions). hCAP-18 is cleaved by serine proteases, similar to those in the kallikrein family, to exhibit specific antimicrobial activity against pathogens (66, 73). Multiple functions of vitamin D-induced LL-37 extend far beyond its direct ability to kill pathogens, support autophagy, enhance phagolysosome fusion, and modulate inflammatory responses (74).

Several factors influence the effectiveness of the vitamin D-dependent LL-37 pathway: vitamin D status in the body [serum 25(OH)D_3_ levels], genetic variations in VDR and related enzymes, epigenetic regulation (such as DNA methylation and histone acetylation), and interactions with metabolites, such as short-chain fatty acids from the gut microbiome (34, 75).

Resident cells in the skin, such as keratinocytes, sebocytes, eccrine gland cells, and mast cells, produce and secrete AMPs. Immune cells, such as neutrophils and NK cells, contribute to the pool of AMPs in the skin. *In vivo* experiments on human skin have demonstrated that the topical application of calcipotriol, a vitamin D analog, increases the expression of hCAP18/LL-37 during wounding, preventing infection (76). 1,25(OH)_2_D_3_ acts in human keratinocytes through a complex signaling pathway involving AP-1, p38-induced PPAR-*γ* and MAPKs for the expression of hCAP18 and hBD-3 (77). Studies by Durnaś *et al*. and Thijs *et al*. revealed that vitamin D promotes the expression of human neutrophil peptides (HNPs), particularly HNP-1. 1,25(OH)_2_D_3_ upregulates HNP1-3 in neutrophils, increasing their ability to kill pathogens in a dose-dependent manner (78, 79).

### 4.2 Vitamin D3 as a direct antifungal compound: translational feasibility and safety

In addition to host immunomodulation, several *in vitro* and preclinical studies have investigated whether unmodified vitamin D3 can behave as a direct antifungal compound against *Candida* or *Cryptococcus* (80–83). This question should be kept conceptually distinct from vitamin D-dependent AMP induction. The direct-compound observations are mechanistically interesting, but the reported inhibitory exposures are often in the μg/mL range, which is supraphysiologic relative to normal circulating vitamin D metabolite concentrations and should not be assumed to be safely reproducible as free systemic drug levels (32, 80, 83–85). Because vitamin D3 is a lipophilic prohormone that is subject to distribution, protein binding, metabolic conversion, and calcium-phosphate regulatory toxicity, simple extrapolation from *in vitro* growth inhibition to clinically useful systemic antifungal therapy is not justified (84–86). For this reason, any direct antifungal interpretation should be treated as a pharmacological development question rather than as evidence that routine vitamin D supplements function as antifungal drugs.

### 4.3 Tiered evidence for vitamin D-related antifungal claims

To separate biological plausibility from clinical inference and to avoid conflating host immunomodulation with direct-compound activity, the antifungal literature discussed below is organized into mechanistic evidence, animal model evidence, observational human evidence, and randomized trial evidence. Clinical claims are restricted to the latter two tiers, whereas direct antifungal compound claims are interpreted with additional attention to exposure feasibility and safety. Table 1 condenses the vitamin D-related evidence tiers for rapid comparison.

**Table 1:** Summary of vitamin D-related antifungal evidence.

Evidence tier	Main fungus-relevant findings summarized in this review	Translational interpretation
Mechanistic	Human epithelial and myeloid studies support vitamin D-dependent CAMP induction and, under inflammation, DEFB4 regulation. Separate *in vitro* studies report direct vitamin D3 activity against Candida or Cryptococcus, usually at supraphysiologic concentrations or under simplified conditions (66, 71, 72, 80–83, 86–88).	Supports biologic plausibility, not clinical efficacy.
Animal model	Murine aspergillosis and candidiasis models support *in vivo* plausibility, but responses vary with vitamin D form and dose. Higher-dose harm in systemic Candida models and rodent CAMP limitations temper inference (82, 90–93).	Useful for *in vivo* signal detection, but not sufficient for therapeutic recommendation.
Observational human	Direct human fungal evidence is limited mostly to associations, such as lower vitamin D status with greater oral Candida colonization or reduced ex vivo candidacidal activity in selected populations (95, 96).	Hypothesis-generating only; confounding and reverse causation remain possible.
Randomized/interventional	No randomized trials with fungal clearance, mortality, or other fungal endpoints were identified. Existing intervention studies mainly show AMP induction or barrier effects in nonfungal settings (97, 98).	Clinical antifungal benefit remains unproven.

Studies on human macrophages and epithelial/myeloid cells at the host level have shown that TLR2 activation and 1,25(OH)_2_D_3_ signaling induce CAMP transcription and, under inflammatory conditions, DEFB4 expression, supporting a vitamin D-dependent AMP axis (87, 88). In contrast, the direct-compound literature shows that vitamin D3 can inhibit *Candida* hyphal growth and biofilm formation and impair *Cryptococcus neoformans* biofilm formation and cell wall integrity *in vitro* (80, 81, 83). These pathogen-level findings establish mechanistic interest, but they do not by themselves show that clinically achievable systemic vitamin D exposure will reproduce antifungal drug concentrations at infection sites (32, 84–86). The relevance of 25(OH)D_3_ remains mechanistically important because it provides a substrate for local generation of the active hormone and downstream AMP responses (89).

### 4.4 Evidence from an animal model

In murine aspergillosis, vitamin D deficiency impairs pulmonary resistance and alters inflammatory and autophagic responses (90–92). In invasive candidiasis models, vitamin D3 has been reported to reduce fungal burden (82), but a separate systemic *Candida* study demonstrated a bimodal dose response, with benefits at lower doses and worse outcomes at higher doses (93). These animal studies are informative primarily for host-level immunomodulation and for dose-related caution; however, because rodent cathelicidin regulation does not recapitulate the architecture of the primate CAMP VDRE, they should not be interpreted as direct proof of a human vitamin D-driven LL-37 mechanism or of safe, clinically feasible direct antifungal dosing (71, 72, 94).

### 4.5 Observational human evidence

Direct evidence related to human *Candida* remains limited and is largely observational or translational. A lower vitamin D3 status has been associated with greater oral *Candida* colonization in psoriasis cohorts (95), and patients with hereditary resistance to 1,25(OH)_2_D_3_ presented reduced *ex vivo* neutrophil killing of *C. albicans* despite preserved phagocytosis (96). These data support association and biological plausibility for host defense, but they do not establish that vitamin D supplementation improves clinical fungal outcomes or that vitamin D3 behaves as a direct antifungal drug in humans.

### 4.6 Randomized trial evidence

Direct randomized trials for candidiasis or other fungal endpoints are essentially lacking. In the literature cited here, vitamin D supplementation increased AMP activity in airway surface liquid in healthy nonsmokers (97) and increased LL-37 in patients with Crohn’s disease in remission (98); however, these studies were not fungal infection trials. Additional human studies in dental, urinary, intestinal, and metabolic contexts (99–101) may inform broader mucosal or AMP biology, but they should not be used as evidence of antifungal clinical efficacy or direct antifungal pharmacotherapy (102, 103). The older three-patient chromoblastomycosis case series (104) is hypothesis-generating only and does not alter the conclusion that the randomized clinical landscape for fungal disease remains very limited.

The accumulated evidence regarding vitamin D consistently identifies antimicrobial peptides as potential downstream effectors; however, this evidence alone does not clarify the mechanisms by which these peptides affect fungal cells. Consequently, the focus of the following section shifts from vitamin D-centered interpretation to an examination of the fungus-targeted mechanisms and translational behavior of LL-37 and defensins.

## 5 Antifungal mechanisms of AMPs

**Figure 3:**
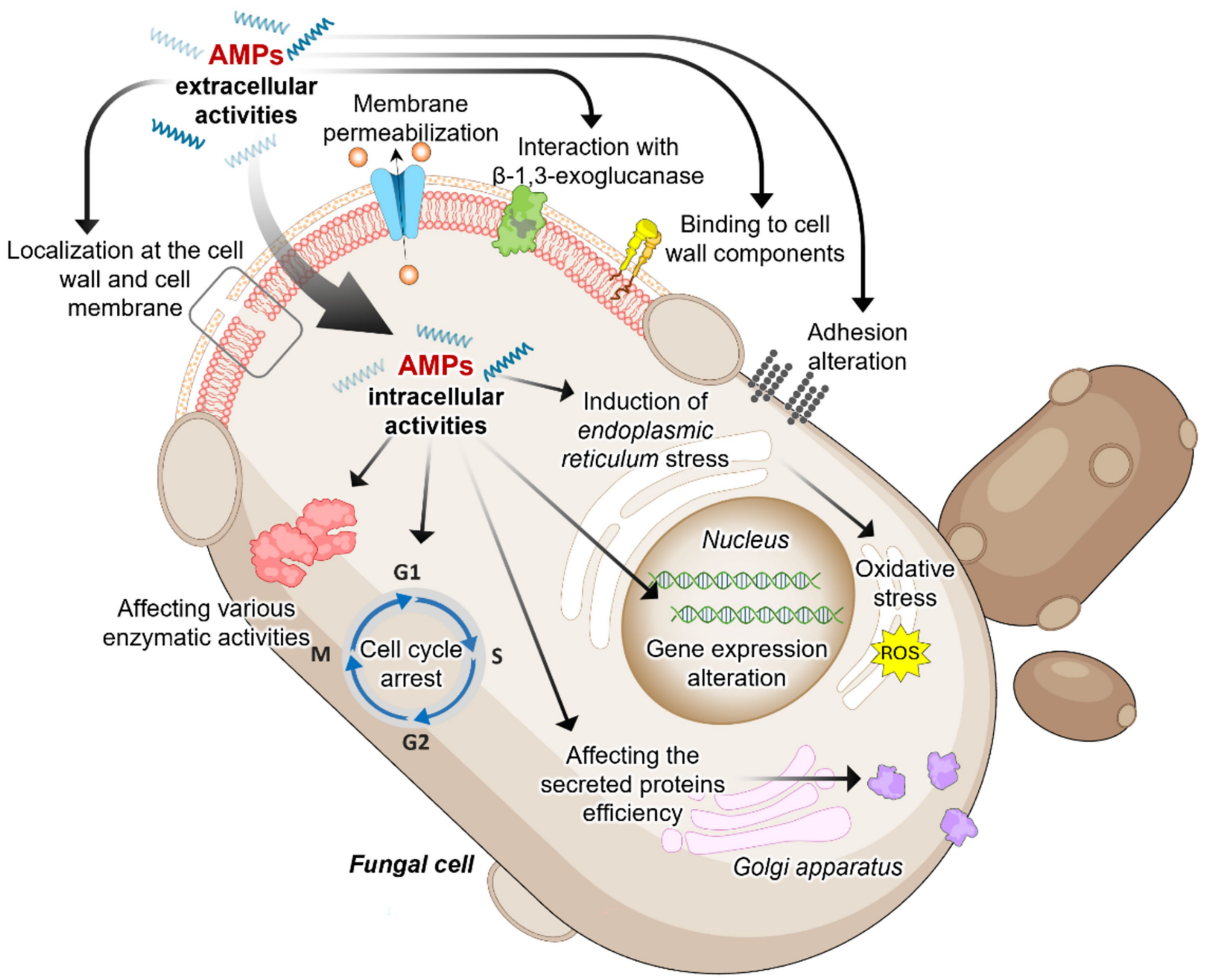
Mechanisms of the antifungal action of AMPs. ROS, Reactive oxygen species. See further details in the text.

As described above, AMPs exert growth-inhibiting and/or growth-killing effects on a variety of fungal pathogens. Most of the evidence summarized in this section is mechanistic and preclinical, and it should therefore be interpreted as supporting biological plausibility rather than established clinical efficacy. These AMP-mediated antifungal mechanisms are conceptually distinct from claims that native vitamin D3 itself functions as a direct antifungal drug. *C. albicans* is a widely used model organism for studying the antifungal effects of hBD-1-3 and LL-37 (105). The accumulation of experimental data on *C. albicans* now allows investigation of how AMPs disrupt the fungal plasma membrane and cell wall and adhesion to host cells, providing molecular-level insights into pathogen–host interactions and potential therapeutic strategies against fungal infections (106, 107). The spectrum of both extracellular and intracellular mechanisms through which AMPs target fungal pathogens is shown in Figure 3.

The mechanisms of hBDs can involve oxidative stress, cell cycle arrest, increased membrane permeability, and ATP efflux in fungal cells (108). Some studies have reported that bBDs can act synergistically with other AMPs and are sensitive to salt concentration, which can influence their candidacidal activity (109, 110). The key features of the antifungal mechanism of action of LL-37 can be described as follows: it primarily targets the fungal cell wall by interacting with components such as *β*-1,3-exoglucanase, causing a reduction in cell wall thickness and polysaccharide content and thus interfering with cell wall reconstruction and integrity (111, 112). Moreover, LL-37 interacts directly with the fungal plasma membrane, causing structural damage and rapid membrane permeabilization (78, 113). In addition to its direct antimicrobial effects, LL-37 is a pleiotropic molecule that can enter host cells and modulate the expression of a wide array of genes involved in key cellular processes (114). In fungal cells, exposure to LL-37 leads to multiple harmful effects, including cell cycle arrest, oxidative stress caused by elevated reactive oxygen species, and disruption of the ability of the endoplasmic reticulum (ER) to maintain protein balance. The observed activation of autophagy-like structures may be a cellular attempt to cope with damage or a sign of cellular dysfunction (115).

### 5.1 Antifungal activities of hBD

*β*-Defensins exhibit antifungal activity against various *Candida* species, including *C. albicans, C. krusei, C. tropicalis,* and *C. parapsilosis*. The multifaceted fungicidal mechanism of hBDs involves direct disruption of fungal cell membranes, cell cycle arrest in the G0/G1 phase, induction of oxidative stress by altering antioxidant enzyme activity, and potential synergistic interactions with other antimicrobial agents (109, 116). Studies by Joly *et al*. have shown that hBD-2 and hBD-3 require Ssa1/2 (a member of the heat shock 70 protein family) on the surface of *C. albicans* for fungicidal activity, leading to membrane permeabilization and cell death (117). Argimon *et al*. demonstrated that *C. albicans* responds to hBD-2 and hBD-3 by activating its high-osmolarity glycerol pathway, a key signaling mechanism for adapting to osmotic stress (118). This activation triggers a complex response, including changes in gene expression and metabolism, such as increased glycerol synthesis, to help the fungus survive high-osmolarity environments.

Although both hBD-2 and hBD-3 bind to Ssa1/2, their killing mechanisms are energy dependent and are similar to those of salivary histatin 5 (Hst 5) (119). Moreover, hBD-3 can increase the activity of *β*-1,3-exoglucanase (Xog1) in *C. albicans*, thereby reducing the adherence of the fungus to abiotic surfaces, such as plastic (107, 120, 121). At a concentration of 10 μM, hBD-3 significantly reduced *C. albicans* growth, resulting in a 67.9% decrease in the number of CFUs (107). Like hBD-2 and hBD-3, hBD-1 can destabilize the membrane of *C. albicans*, causing cell death (109, 122). Reducing the number of disulfide bridges in hBD-1 increases its antifungal activity against *C. albicans*, resulting in its transformation into a more potent peptide, as disulfide bonds normally inhibit its antimicrobial function (123).

Epithelial defense is a crucial part of the innate immune system and acts as the first line of defense against fungal pathogens at the epithelial barrier. It has also been reported that hBD-2 and hBD-3 upregulate the expression of tight junction proteins and increase transepithelial electrical resistance, which strengthens the barrier and reduces the invasive ability of *C. albicans* (124, 125). Caco-2 cells, along with oral and bronchial epithelial cells, produce hBD-2 when exposed to fungi, creating a first line of defense against pathogens such as *C. albicans, C. krusei, C. tropicalis*, and *C. parapsilosis* (126). The presence of *C. albicans* significantly increases the expression of hBD-2 and hBD-3 in the esophagus and oral mucosa, whereas hBD-1 expression remains relatively unchanged or only slightly increases, suggesting a differential role for these defensins in host defense against fungal infection (127, 128). Infection of gingival epithelial cells by *C. albicans* and *C. famata* increases the production of hBD-1, hBD-2, and hBD-3 (129, 130). In their studies, Rizzo et al. used HeLa cells as an *in vitro* model to show how *L. crispatus* modulates the host’s innate immune response by expressing hBD-2 and hBD-3 to combat *C. albicans* infection (131). This antifungal activity is also exhibited by epithelial cells in the respiratory tract (132, 133). During *Aspergillus* infection, respiratory epithelial cells detect the fungus, leading to increased expression and secretion of defensins, such as hBD-2 and hBD-9 (134).

The fungicidal mechanism of *α*-defensins has been studied less extensively than that of *β*-defensins, although both are AMPs known to disrupt fungal cell membranes. HNP-1 kills *C. albicans* by depleting intracellular ATP and increasing extracellular ATP, a mechanism that does not depend on the fungal proteins Ssa1/2, unlike the action of hBD-2 and hBD-3, which require these fungal proteins for their antifungal activity (119). Research has confirmed that the secretion of the human α-defensin HNP1-3 is significantly increased in both whole blood and esophageal tissue following exposure to *C. albicans* (126, 135). Edgerton et al. demonstrated that HNP-1 and salivary histatin 5 (Hst 5) kill *C. albicans* via similar mechanisms, characterized by nonlytic ATP efflux at comparable active concentrations and magnitudes, although the exact cellular targets remain unclear (136). In a study by Chairatana *et al*., HD6 was shown to prevent *C. albicans* from adhering to and invading human intestinal epithelial cells and to suppress biofilm formation (137).

### 5.2 Antifungal activities of LL-37

The net positive charge and amphipathic *α*-helical structure allow LL-37 to bind to negatively charged microbial membranes, leading to membrane disruption and cell death. Permeabilization is a key mechanism through which LL-37 kills microbial pathogens (138, 139). According to several studies, LL-37 disrupts microbial membranes by forming transmembrane pores and can also affect lipid vesicles, causing leakage of their contents (140–142). In *Candida* cells, LL-37 induces severe membrane disruption, leading to disintegration into vesicle-like structures approximately 100 nm in size (143).

LL-37 is found on skin and mucosal surfaces, such as the respiratory tract and oral cavity, at concentrations of approximately 2 to 5 μg/mL under healthy conditions. At sites of inflammation, these levels can increase significantly (e.g., up to 30 μg/mL). LL-37 is crucial for maintaining mucosal immunity and acts both as a direct antimicrobial agent and as a modulator of immune responses (144). The gene expression and secretion of LL-37 in keratinocytes are upregulated when the cells are exposed to the *C. albicans* cell wall phospholipomannan (145). Tsai et al., using a spot assay and a FUN-1 assay that incorporated flow cytometry, demonstrated that LL-37 has a dose-dependent candidacidal effect at concentrations of 20 μg/mL and higher (146). Another study by López-García *et al*. in a mouse model revealed that while cathelicidins can kill *C. albicans* and inhibit its growth by disrupting its membrane, the induction of LL-37 during *C. albicans* infection did not lead to systemic or subcutaneous resistance. Instead, they appeared to work best in the ionic environment of sweat, where the natural processing of LL-37 enhances its antifungal activity (147). Indeed, several studies have confirmed that LL-37 can be naturally proteolytically cleaved into smaller, more potent peptide fragments, including those of RK-31 and KS-30. This process occurs on the skin surface and yields peptides with enhanced activity against *Candida* (147, 148).

LL-37 also induces vacuole expansion and membrane permeabilization in planktonic *C. albicans* cells, causing rapid ATP efflux and, ultimately, fungal cell death. This effect is linked to the disruption of the fungal plasma membrane and its subsequent impact on calcium homeostasis, which can also involve the vacuole (149, 150). Moreover, LL-37 triggers the unfolded protein response (UPR) pathway in *C. albicans* to cope with endoplasmic reticulum (ER) stress and leads to the generation of ER-derived ROS (151). Similarly, LL-37 can cause oxidative stress and cell cycle arrest in *C. auris* cells at concentrations ranging from 25 to 100 μg/mL for inhibition and 50 to 200 μg/mL for killing (115).

Another important function of LL-37 is its ability to reduce the adhesive capacity of *C. albicans* by binding to its cell wall carbohydrates, such as mannan, altering the cell surface and leading to aggregation (146). Scarsini *et al*. reported that 64 μM LL-37 solution can inhibit the initial adhesion of *Candida* cells to surfaces such as polystyrene and silicone and can impede their ability to form biofilms. However, the same study also reported that LL-37 was ineffective against *Candida* biofilms that had already formed (152). In addition to its antifungal effects on *Candida* species, LL-37 inhibits *Aspergillus fumigatus* by directly attacking the fungus and preventing an excessive inflammatory response (153). Table 2 presents a comparative summary of the AMP literature, distinguishing between the extensive mechanistic data and the relatively limited animal, observational, and clinical evidence.

**Table 2:** Hierarchical summary of AMP antifungal evidence and its translational implications.

Evidence tier	Main fungus-relevant findings summarized in this review	Translational interpretation
Mechanistic	Most AMP antifungal evidence is cell-based and heavily Candida-focused. LL-37 and defensins can disrupt fungal membranes or cell walls, induce ATP efflux, oxidative or ER stress, and reduce adhesion or early biofilm formation (107, 108, 122, 123, 138, 139, 141).	Strong pathway-level support for antifungal activity.
Animal model	Compared with the mechanistic literature, direct *in vivo* AMP evidence is limited. Available preclinical work suggests context-dependent activity of cathelicidins or their processed fragments and possible relevance in aspergillosis, but efficacy remains model specific (147, 153).	Suggestive preclinical support, but still not therapeutic proof.
Observational human	Human epithelial, mucosal, blood, and tissue studies show induction of defensins and LL-37 during fungal exposure, supporting host-response relevance at barrier sites (126–129, 145).	Demonstrates biologic response in humans, not treatment benefit.
Clinical/interventional	AMP-directed antifungal trials are essentially absent in the literature summarized here. Delivery, proteolytic degradation, salt sensitivity, and stability or toxicity barriers remain major translational obstacles.	Clinical efficacy remains unestablished.

This dual mechanism supports further preclinical investigations in aspergillosis, particularly in immunocompromised settings. Collectively, these mechanistic findings regarding AMPs establish a biological rationale for translational research. However, they also emphasize that activity observed *in vitro* or in animal models does not necessarily predict clinical efficacy in antifungal interventions. Consequently, the final section addresses the primary interpretive, pharmacologic, and model-related limitations that influence the interpretation of these data.

## 6 Model constraints, antifungal feasibility, safety, and knowledge gaps

For antifungal strategies involving vitamin D, it is important to distinguish two different translational propositions: first, vitamin D is a host-directed immune modulator that may influence AMP expression and mucosal defense; second, vitamin D3 is a directly administered antifungal compound. The first proposition is biologically plausible but still only weakly supported by fungal clinical trial data, whereas the second faces additional pharmacologic and toxicologic barriers beyond the model limitations discussed above.

### 6.1 Primate-specific regulation of the CAMP VDRE and implications for rodent models

The widely cited vitamin D-to-LL-37 narrative is mechanistically strongest in humans and other primates because the CAMP promoter contains a functional VDRE embedded within a primate-specific AluSx short, interspersed element. This promoter architecture is not conserved in mice, rats, or dogs (66, 71, 72). Accordingly, direct vitamin D-induced CAMP/LL-37 transcription should be viewed as a primate-enriched mechanism rather than a universally conserved mammalian response.

Rodent fungal infection models therefore remain useful for studying the broader effects of vitamin D on inflammation, autophagy, barrier responses, and fungal burden, but they are imperfect proxies for the specific human vitamin D-to-LL-37 axis (90–93). Even human CAMP transgenic mice only partially address this limitation: topical 1,25(OH)_2_D_3_ can induce human transgenes in the skin, yet macrophages do not fully reproduce the TLR-25(OH)D_3_-CYP27B1-CAMP response observed in human cells because upstream Cyp27b1 regulation differs (94). This is why the rodent data in this review are interpreted as supportive preclinical evidence, not as direct proof of human LL-37-mediated clinical benefit.

### 6.2 Direct antifungal feasibility and safety constraints

The direct antifungal hypothesis faces a major exposure feasibility problem. The concentrations reported to inhibit Candida or Cryptococcus *in vitro* are frequently in the μg/mL range (80, 81, 83), whereas vitamin D physiology and clinical supplementation are centered on much lower circulating metabolite concentrations under tight endocrine control (32, 84, 85). Because vitamin D3 is fat soluble, highly distributed, and metabolically converted before endocrine activity is expressed, *in vitro* inhibition at high nominal concentrations should not be assumed to translate into achievable free antifungal concentrations at sites of invasive infection (86).

Hypercalcemia and hyperphosphatemia are central systemic safety constraints and are not minor caveats. Increased vitamin D exposure to direct antifungal concentrations is associated with calcium-phosphate dysregulation, nephrocalcinosis, and related toxicity (84, 154). This concern is especially important in granulomatous fungal diseases, such as disseminated histoplasmosis, where activated macrophages may already increase extrarenal 1,25(OH)_2_D production and predispose patients to vitamin D-mediated hypercalcemia (86). In such settings, supplementation cannot be treated as a benign adjunct and may worsen the metabolic complications of infection.

Dose-dependent responses in infection models reinforce this narrow therapeutic window. A 2015 study by Lim *et al*. on systemic Candida infection in mice revealed that while a low dose of active vitamin D improved survival, higher doses led to worse outcomes (93). This pattern does not meet the same threshold in humans, but it strengthens the argument that more vitamin D is not necessarily better and that toxicology must be central to translational planning.

There is limited direct evidence from clinical trials. However, randomized trials in humans that directly test vitamin D as an adjunctive therapy for fungal infections are lacking. The interventional studies cited in this field mainly assess AMP induction or barrier-related outcomes in nonfungal settings rather than mycologically confirmed fungal endpoints (97, 98). Even within the host-modulatory framework, excessive activation of the vitamin D-cathelicidin axis may be context dependent and requires careful interpretation (155). This pattern is consistent with recent infectious disease reviews, which report that observational associations between lower vitamin D status and worse outcomes are generally stronger and more reproducible than randomized trial evidence is, whereas heterogeneity in baseline deficiency, dosing regimens, and clinical endpoints remains a major barrier to causal inference (156).

Although the vitamin D-cathelicidin axis is often considered protective, it must remain tightly regulated (155). Imbalances can contribute to harmful excessive inflammation in specific tissues or disease settings, which further argues against the assumption that progressively increased vitamin D exposure will produce uniformly beneficial antifungal effects.

### 6.3 Knowledge gaps persist

Mechanisms beyond AMPs. The full scope of the antifungal action of vitamin D is likely broader than that of AMP. For example, vitamin D is known to promote autophagy, which can be involved in host defense against pathogens such as *A. fumigatus* (91). More research is needed to understand the relative contributions of different mechanisms.

1. Adaptive fungal responses. The adaptive response of *C. albicans* to counteract the inhibitory effects of vitamin D3 is evidenced by the increased expression of the ALS1, SAP6, and EFG1 genes following exposure to vitamin D3 (81). These changes may reflect fungal survival strategies under vitamin D-induced stress. In addition, *Candida albicans* can directly counter AMP activity; for example, secreted aspartyl proteases can cleave LL-37 and attenuate its antifungal and immunomodulatory functions, highlighting fungal evasion as a key translational gap (107, 157).
2. Delivery and stability of AMP therapeutics. Translating AMP biology into therapy remains challenging because candidate peptides may be degraded by host or fungal proteases, inactivated by physiologic salt or protein binding, cleared rapidly, or cause local/systemic toxicity. Future work should therefore address peptide engineering, formulation, controlled delivery, and tissue-specific exposure rather than assume that *in vitro* activity will be retained *in vivo* (112, 158).
3. Clinically meaningful endpoints in interventional studies. Future vitamin D- or AMP-based intervention trials should move beyond surrogate markers such as serum 25(OH)D levels, AMP expression, or *ex vivo* candidacidal activity and instead prioritize fungal clearance/mycological response, mortality, ICU or hospital length of stay, organ dysfunction, and rigorous safety assessment.
4. Data for certain pathogens are limited. While some data exist for vitamin D and AMPs against *Candida* and *Aspergillus*, research on other clinically significant fungal pathogens, such as *Cryptococcus* species, is limited. The specific roles and effectiveness of AMPs against these and other fungi remain underexplored.

## 7 Conclusion

In alignment with current global fungal priority frameworks and resistance surveillance, this review highlights *Candida* spp., *Aspergillus fumigatus*, and *Cryptococcus neoformans*. Resistant dermatophytes are also considered, although this area remains less developed translationally. These findings indicate that vitamin D functions primarily as a biologically plausible host-directed modulator of antimicrobial peptide responses rather than as a validated antifungal therapy. The strongest evidence exists at the mechanistic and animal model levels, whereas human data related to *Candida* are predominantly observational or translational. Randomized trials with fungal clinical endpoints are limited. Therefore, no direct therapeutic recommendations or broad clinical extrapolations can be made at this time. Future research should focus on WHO-priority pathogens, incorporate resistance-aware study designs, and utilize clinically meaningful endpoints such as fungal clearance, mortality, ICU length of stay, and organ dysfunction. The accompanying tiered summary tables emphasize that translational claims should rely primarily on the limited human and interventional evidence, which is not inferred from the more extensive mechanistic literature.
